# Vascular α1A Adrenergic Receptors as a Potential Therapeutic Target for IPAD in Alzheimer’s Disease

**DOI:** 10.3390/ph13090261

**Published:** 2020-09-22

**Authors:** Miles Frost, Abby Keable, Dan Baseley, Amber Sealy, Diana Andreea Zbarcea, Maureen Gatherer, Ho Ming Yuen, Matt MacGregor Sharp, Roy O. Weller, Johannes Attems, Colin Smith, Paul R. Chiarot, Roxana O. Carare

**Affiliations:** 1 Faculty of Medicine, University of Southampton, Southampton SO16 6YD, UK; mrjf1g15@soton.ac.uk (M.F.); a.c.keable@soton.ac.uk (A.K.); dan@baseley.org.uk (D.B.); as4e15@soton.ac.uk (A.S.); daz1g16@soton.ac.uk (D.A.Z.); m.gatherer@soton.ac.uk (M.G.); H.M.Yuen@soton.ac.uk (H.M.Y.); m.t.sharp@soton.ac.uk (M.M.S.); row@soton.ac.uk (R.O.W.); 2 Translational and Clinical Research Institute, Newcastle University, Newacstle upon Tyne NE4 5PL, UK; johannes.attems@ncl.ac.uk; 3 Centre for Clinical Brain Sciences, University of Edinburgh, Edinburgh EH16 4SB, UK; Col.Smith@ed.ac.uk; 4 Department of Mechanical Engineering, State University of New York at Binghamton, Binghamton, NY 13902, USA; pchiarot@binghamton.edu

**Keywords:** α_1A_ adrenergic receptor, intramural periarterial drainage, cerebral blood vessels

## Abstract

Drainage of interstitial fluid from the brain occurs via the intramural periarterial drainage (IPAD) pathways along the basement membranes of cerebral capillaries and arteries against the direction of blood flow into the brain. The cerebrovascular smooth muscle cells (SMCs) provide the motive force for driving IPAD, and their decrease in function may explain the deposition of amyloid-beta as cerebral amyloid angiopathy (CAA), a key feature of Alzheimer’s disease. The α-adrenoceptor subtype α_1A_ is abundant in the brain, but its distribution in the cerebral vessels is unclear. We analysed cultured human cerebrovascular SMCs and young, old and CAA human brains for (a) the presence of α_1A_ receptor and (b) the distribution of the α_1A_ receptor within the cerebral vessels. The α_1A_ receptor was present on the wall of cerebrovascular SMCs. No significant changes were observed in the vascular expression of the α_1A_-adrenergic receptor in young, old and CAA cases. The pattern of vascular staining appeared less punctate and more diffuse with ageing and CAA. Our results show that the α_1A_-adrenergic receptor is preserved in cerebral vessels with ageing and in CAA and is expressed on cerebrovascular smooth muscle cells, suggesting that vascular adrenergic receptors may hold potential for therapeutic targeting of IPAD.

## 1. Introduction

Alzheimer’s disease (AD) is the commonest form of dementia, and a key pathological feature of AD is represented by the accumulation of amyloid-beta (Aβ) within the walls of cortical and leptomeningeal arteries as cerebral amyloid angiopathy (CAA) [1]. The changes observed in early CAA are astrogliosis, combined with a dysregulation in lipid metabolism, and Apolipoprotein E and Triggering receptor expressed on myeloid cells 2 (TREM2) [2]. While in familial CAA there is overproduction of normal or mutated proteins such as Aβ, cystatin C or ABri, in sporadic CAA it is the failure of clearance rather than overproduction of Aβ that is responsible for its accumulation in the ageing brain [3,4]. There are several ways in which Aβ can be cleared, such as transcytosis via lipoprotein-related-protein-1 or phagocytosis by perivascular cells or microglia, but these mechanisms fail with ageing [5]. The role of vascular factors such as small vessel disease has been recently recognized in the pathogenesis of CAA and AD [6]. Apart from the supply of blood, which decreases in early AD through hypoperfusion, the cerebral arteries have another recently recognized function: the drainage of waste and soluble interstitial fluid along their walls [5]. Lymphatic drainage of the brain occurs along the basement membranes of capillaries and arteries, as intramural periarterial drainage (IPAD) [7,8,9]. With increasing age and possession of ApoE ε4 genotype, vascular basement membranes change their composition, thereby reducing the efficiency of IPAD [10,11,12].

Multi-scale modelling of cerebral arteries demonstrates that the motive force generated by cerebrovascular smooth muscle cells (SMCs), termed vasomotion, is the motive force for IPAD, and this has been supported by recent experimental work [13,14,15]. Increased levels of myocardin and serum response factor in blood vessels in CAA may reflect a decrease in the function of SMCs and clearance of Aβ [16,17]. Since the SMCs generate the vasomotion for IPAD, the innervation of cerebrovascular SMCs is an attractive target for improving IPAD and subsequently preventing or ameliorating CAA.

Leptomeningeal vessels are innervated extrinsically by the peripheral nervous system, whereas parenchymal vessels are innervated intrinsically from within the brain, receiving afferents from subcortical neurons of the raphe nucleus, nucleus basalis and locus coeruleus (LC). Noradrenaline mediates smooth muscle contraction within blood vessels via α-adrenergic receptors, otherwise known as α-adrenoceptors (α-ARs) [18]. LC, the main noradrenergic nucleus in the brain, degenerates in early stages of AD [19,20,21]. It is known that SMCs express α-Ars, but the expression of α-ARs specifically on SMCs of the cerebral blood vessels is not well documented [22].

In this human neuropathological study, we characterised the expression of α_1A_-AR within the human cerebral vasculature of the occipital lobe, a region preferentially affected by CAA and on cultured human brain vascular SMCs. We hypothesised that α_1A_-AR is preserved in the ageing arteries and in CAA, providing a look to the future for targeting α_1A_-AR for the prevention and treatment of CAA.

## 2. Results

### 2.1. Post-Mortem Delay Does Not Adversely Affect AR Staining

The association between AR expression and post-mortem delay was investigated in grey and white matter. The Pearson’s correlation coefficient showed a positive correlation in the grey matter (n = 15, r = 0.51, *p* = 0.05) and the white matter (n = 15, r = 0.15, *p* = 0.6); however, a sensitivity analysis revealed two outliers, and following removal of these outliers, there was only a weak positive correlation observed in the grey and white matter (0.16 and 0.12, respectively), which was not considered statistically significant (*p* = 0.609 and 0.707 respectively). We concluded that post-mortem delay was therefore unlikely to have a significant impact on the findings reported. As our numbers of cases were low, we included the two outliers in the analysis of the α_1A_-AR. 

### 2.2. The Overall Pattern of Immunocytochemistry for α_1A_-AR on the Cerebral Vessels Is Not Affected by Age

**Figure 1:**
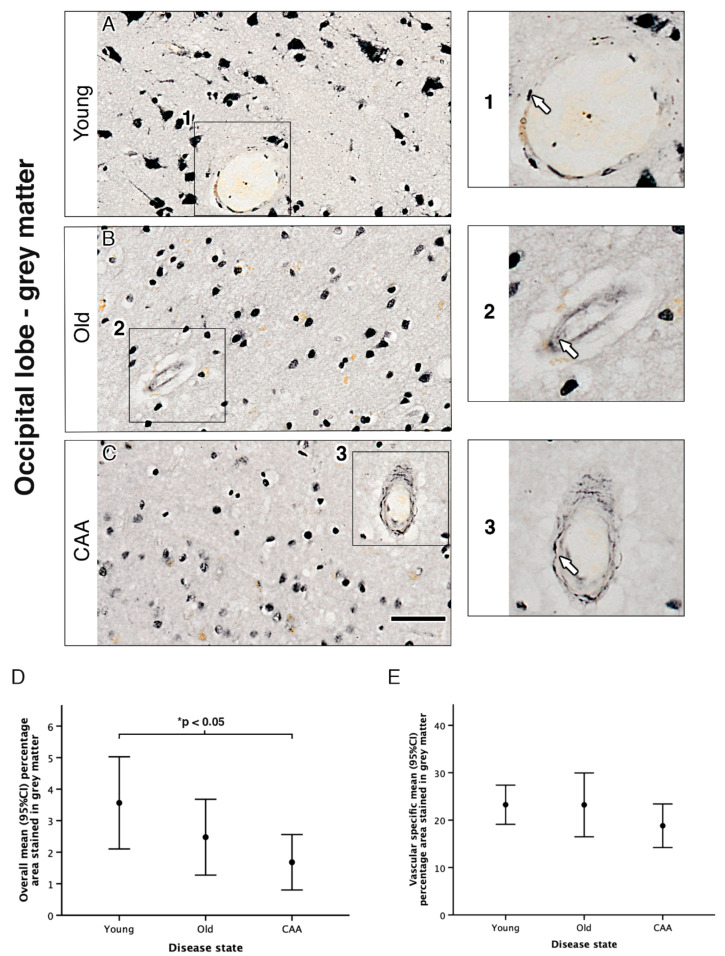
The pattern of distribution of the α-adrenoceptors alpha1a adrenergic receptor (α_1A_-AR) in the grey matter of the occipital lobe. Neuronal staining appeared most intense in young cases (**A**) and decreased in both old (**B**) and cerebral amyloid angiopathy (CAA) cases (**C**). In all cases, α_1A_-AR immunoreactivity was observed in vessel walls (enlarged boxes 1, 2 and 3). There was a significant decrease in α_1A_-AR in CAA cases compared to young groups (**D**) and no differences observed in the vessel wall (**E**). Graphs depict the mean ±95% confidence intervals; *n* = 5. Scale bar 50 µm. * *p* < 0.05.

**Figure 2:**
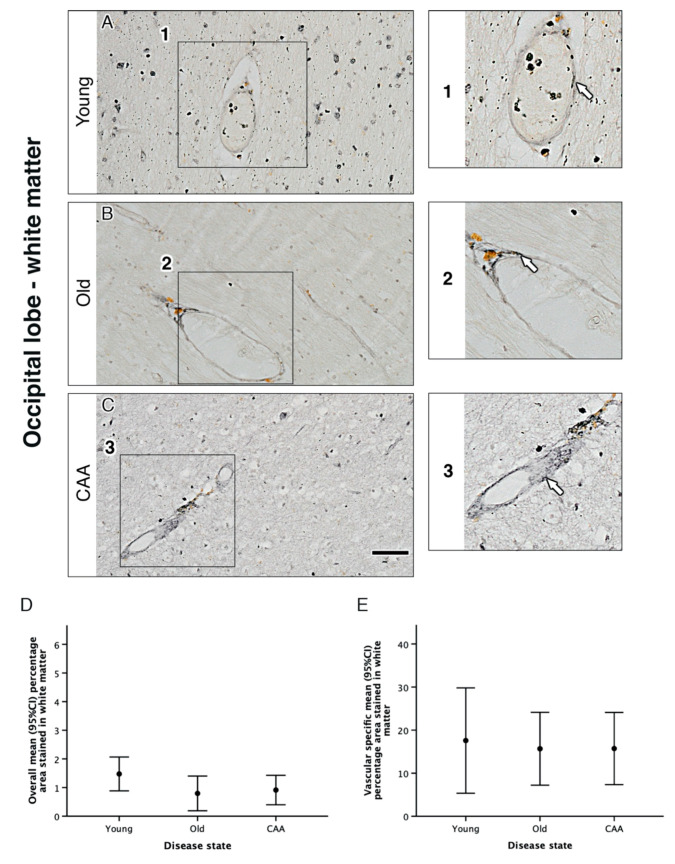
The pattern of distribution of the α_1A_-AR in the white matter of the occipital lobe. Neuronal staining appeared most intense in young cases (**A**) and appeared to decrease in both old (**B**) and CAA cases (**C**). In all cases, α_1A_-AR immunoreactivity was observed in vessel walls (enlarged boxes 1, 2 and 3). There were no significant differences observed in overall percentage area staining (**D**) or the percentage of the vessel wall positive for α_1A_-AR in white matter (**E**). Graphs depict the mean ±95% confidence intervals, *n* = 5. Scale bar 50 µm.

We used immunohistochemistry to assess overall α_1A_-AR expression in grey and white matter in young, old and CAA cases. In all 15 cases, α_1A_-AR expression was observed within neurons of the parenchyma and the vascular wall in both grey and white matter (Figure 1 and Figure 2).

In both grey and white matter, neuronal staining appeared most intense in young cases and decreased in both old and CAA cases. The CAA cases were all diagnosed with Vonsattel scores 2 (moderate, the tunica media is replaced by amyloid and is thicker than normal) or 3 (severe, extensive amyloid deposition with focal wall fragmentation). Due to the low number of cases, it is not possible to perform correlation with the Vonsattel scores, but all CAA cases were moderate/severe.

**Figure 3:**
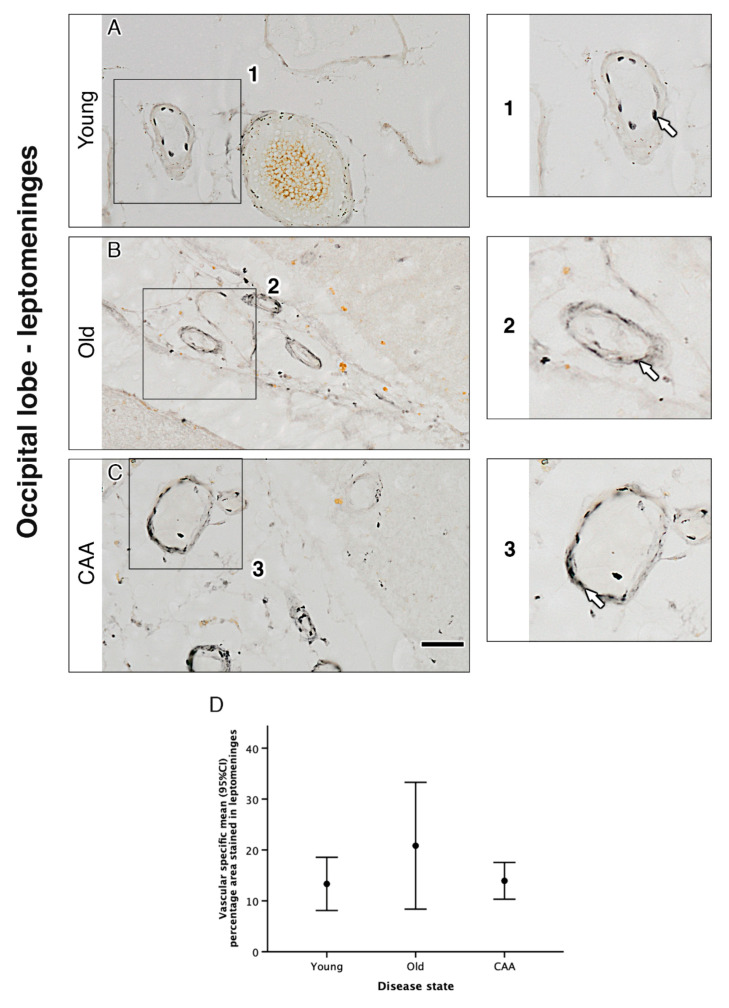
The pattern of distribution of the α_1A_-AR in leptomeningeal vessels of the occipital lobe. In all cases, α_1A_-AR immunoreactivity was observed in vessel walls (**A**, **B** and **C** and enlarged boxes 1, 2 and 3). There were no significant differences in the percentage of the vessel wall positive for α_1A_-AR between cases (**D**). Graph depicts the mean ±95% confidence intervals, *n* = 5. Scale bar 50 µm.

In grey matter, analysis of overall percentage area staining revealed a trend towards a decrease in α_1A_-AR with age and CAA. Overall percentage area stained in young cases was higher than in old cases (3.56% vs. 2.48% *p* = 0.305) and significantly higher than in CAA cases (3.56% vs. 1.68% *p* = 0.029). Overall percentage area stained in old cases was higher but not significantly different from CAA cases (2.48% vs. 1.68% *p* = 0.658) (Figure 1 and Table 1). In white matter, analysis of overall percentage area staining was highest in young cases but not considered statistically significant when compared to old (1.47% vs. 0.79% *p* = 0.113) or CAA cases (1.47% vs. 0.91% *p* = 0.223). There was no significant difference between old and CAA cases (0.79% vs. 0.91%) and no difference between leptomeningeal arteries from young, old and CAA (Figure 2 and Figure 3 and Table 1).

### 2.3. Vascular α_1A_-AR Expression Is Unaltered with Age and Disease in the Occipital Lobe

**Figure 4:**
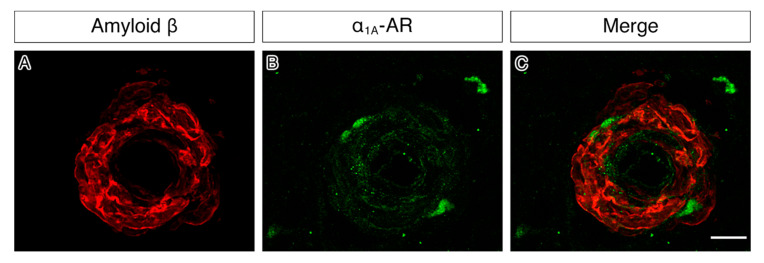
Immunofluorescence for α_1A_-AR (**green**) and Aβ (**red**) on sections of human occipital cortex of a case of CAA. The green immunofluorescence is present within the endothelium and in between the amyloid deposits of the wall of the vessel. Based on the diameter of the vessel and the thickness of the vessel wall, as well as the presence of CAA mainly in cortical arterioles, the vessels are arterioles of 10 µm diameter. Scale bar 25 µm. (**A**) immunostaining for Aβ occupying the whole of the vessel wall in a circumferential manner (**B**) immunostaining for α_1A_-AR showing a focal distribution; (**C**) the overlay image of both Aβ and α_1A_-AR immunofluorescence.

The immunocytochemical expression of α_1A_-AR was observed within the vascular wall in vessels of the grey matter, white matter and the leptomeninges. Vascular staining appeared punctate and dispersed neatly throughout the vessel wall in young cases. This pattern was not observed in the old and CAA cases, where the staining appeared more diffuse (Figure 3). Despite the differences in the appearance of staining, there were no significant differences observed in the percentage area of the vessel wall stained for α_1A_-AR in any of the regions of interest (Table 1). Representative images of α_1A_-AR and amyloid-beta double staining in CAA cases showed α_1A_-AR expression in vessels with a high amyloid load (Figure 4).

### 2.4. α_1A_-AR Co-Localises with Endothelial and Smooth Muscle Cell Markers in Capillaries, Arteries and Veins and with Cultured Vascular Smooth Muscle Cells

**Figure 5:**
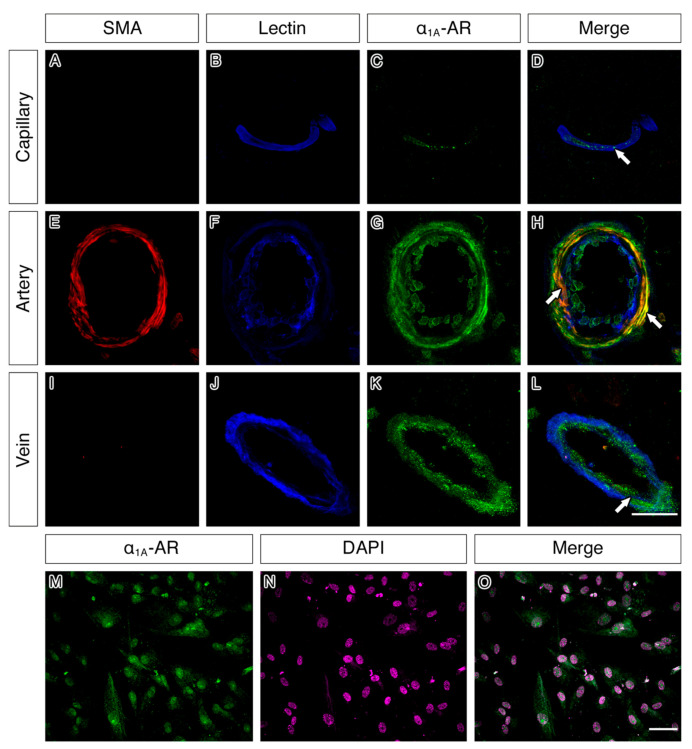
α_1A_-AR immunoreactivity in the vessel walls of capillaries, arteries and veins and within cultured human brain vascular smooth muscle cells. α_1A_-AR immunoreactivity (**green**) was observed colocalised (**white arrows**) to lectin (**blue**) in the vessel walls of capillaries (**A**–**D**), arteries (**E**–**H**) and veins (**I**–**L**) in all cases. α_1A_-AR immunoreactivity was also colocalised with smooth muscle actin (**red**) in arterial walls (**H**) and observed to outline smooth muscle cell bodies in culture (**M**–**O**). Scale bar 50 µm.

Capillaries, arteries and veins all showed expression of α_1A_-AR in the vascular wall in all regions analysed. In the arterial wall, double immunofluorescence revealed a strong co-localisation of α_1A_-AR with smooth muscle actin and to a lesser degree with lectin (Figure 5). A similar co-localisation of α_1A_-AR with lectin was also observed in the vessel walls of capillaries and veins. In cultured human brain vascular smooth muscle cells (HBVSMC), staining for α_1A_-AR appeared to outline the smooth muscle cell body (Figure 5).

## 3. Discussion

This study has shown the qualitative and quantitative differences in the expression of the α_1A_-AR in the grey and white matter of the occipital lobe from young, old and CAA brains. It has also demonstrated that while there is a decrease in overall α_1A_-AR expression in the grey matter of CAA cases, the vascular expression remains relatively unchanged and the α_1A_-AR was still detected in the walls of vessels laden with Aβ.

Changes in AR expression have been previously reported in brain regions affected by AD pathology such as increases in the prefrontal cortex, hippocampus and amygdala. This suggests a compensatory mechanism to overcome a decrease in noradrenergic input due to the degeneration of the locus coeruleus seen in early stages of AD [19,23]. We did not observe any significant quantitative changes to α_1A_-AR expression within the vessels. However, the change in appearance of expression from punctate in young brains to diffuse in old and CAA brains may suggest a redistribution of the α_1A_-AR on the blood vessels, likely resulting in functional vasomotor deficit. This would be particularly relevant to the accumulation of protein deposits in the vessel wall as CAA caused by a reduced efficiency of IPAD, a process that most likely relies upon adrenergic innervation [13].

Preservation of vascular α_1A_-ARs shown in this study highlights their potential as possible therapeutic targets for IPAD in AD, and this requires experimental in vivo testing. Prazosin, the only clinically available α_1_-AR antagonist that can cross the blood–brain barrier has been shown to induce an anti-inflammatory response in the brain, preventing memory deficits over time in mutant transgenic APP23 mice [24]. Furthermore, administration of Prazosin improved behavioural symptoms in patients with agitation/aggression in AD [25]. It remains to be seen whether the effects of Prazosin are mediated by vascular α_1A_-ARs. The morphological relationship between α_1A_-AR immunostaining and Aβ would be a useful exercise in a future in vivo experimental study where we would administer α_1A_-AR antagonist such as Prazosin to test whether it alleviates the pathological features of CAA. *Prazosin for Disruptive Agitation in Alzheimer’s Disease (PEACE-AD)* is a Phase IIb multicenter, randomized, double-blind, placebo-controlled trial of 12-weeks treatment with Prazosin in Alzheimer’s disease, due to end on 31 December 2021 (NCT03710642), and this may reveal evidence for the prevention of CAA or brain haemorrhages. 

The synergistic effects of the other receptors may have profound implications on the pathogenesis of AD and CAA. For example, the vasoconstriction generated by agonists of the α_1A_-ARs is enhanced by stimulation of the apelin receptor [26]. Genetic deficiency of the α_2A_-AR significantly reduces Aβ generation, whereas stimulation of this receptor enhances it [27]. Recent studies also demonstrate that Aβ oligomers bind to the α_2A_-AR, leading to glycogen synthase kinase 3β (GSK3β) hyperphosphorylation of tau [28].

There are limitations to this study, as it is a simple neuropathological assessment of the pattern of distribution of α_1A_-ARs in the vasculature of young, old non-demented and CAA cases, using a small sample size. However, the present study paves the way for experimental targeting of adrenergic receptors for the prevention and treatment of CAA, as we show they are still present in aged and CAA vessels. Further experimental studies testing the effect of adrenergic antagonists on vascular SMCs possibly used in combination with treatments that alter cholinergic innervation will act upon IPAD pathways for the prevention and treatment of CAA and AD.

## 4. Materials and Methods

### 4.1. Brain Tissue Cohort

Sections of 10 μm thickness of post-mortem human occipital cortex were used in this study. Human tissue from young post-mortem donors (≤60 years old) was supplied by the Medical Research Council-funded Edinburgh Sudden Death Tissue Brain Bank (Ethics REC 16/ES/0084). Tissue from old (≥65 years old) and CAA-affected post-mortem donors was supplied by the Newcastle Brain Tissue Resource (Ethics REC 08/H0906/136 + 5). The demographics of the cases used are summarised in Table 2. The cases from the MRC Sudden Death Brain and Tissue Bank (Edinburgh) had no neurological disease during life and no significant neuropathological changes postmortem. CAA cases were diagnosed post-mortem by JA, and Vonsattel scores are included in Table 2.

### 4.2. Cell Culture

Human brain vascular smooth muscle cells (HBVSMC) were obtained from Sciencell (sc-1100). Cells were maintained in a humidified atmosphere of 5% CO_2_ and 95% air at 37 °C in a smooth muscle basal medium (sc-1101b) supplemented with smooth muscle cell growth supplement (sc-1152), 100 U/mL penicillin, 100 μg/mL streptomycin (sc-0503) and 2% foetal bovine serum (sc-0010). The medium was refreshed every 2–3 days according to the manufacturer’s instructions. Cells were plated onto poly-L-lysine coated coverslips in a 24-well plate at a seeding density of 0.05 × 10^6^ cells per coverslip.

### 4.3. Immunohistochemistry on Human Tissue

Sections were deparaffinised and rehydrated through xylene and a graded series of alcohols. Endogenous peroxidase activity was quenched with 3% hydrogen peroxide for 15 min. Sections were washed three times with phosphate-buffered saline (PBS). Heat-induced antigen retrieval was then performed by microwaving in citrate buffer (10 mM, pH 6) for 25 min. Sections were incubated in a blocking solution containing 15% normal goat serum (Sigma, G9023) diluted in phosphate-buffered saline with 0.1% triton X-100 (PBSt) for 1 h prior to overnight incubation at 4 °C with anti α_1A_-AR primary antibody (rabbit polyclonal, 1:200, Protein Tech, 19777-1-AP) diluted in PBSt. After washing with PBS, sections were incubated for 1 h with a biotinylated goat anti-rabbit secondary antibody (Vector BA-1000). Sections were washed with PBS and incubated with Vectastain Avidin-biotin complex (Vector PK-4000) for 1 h. Sections were rinsed with 0.1M sodium acetate buffer, then incubated with glucose oxidase diaminobenzidine nickel solution for 7 min. Finally, sections were rinsed with 0.1M sodium acetate buffer, followed by PBS before dehydration through graded alcohols and xylene and mounted with DPX. Care was taken in the selection of primary antibody to ensure thorough validation had been carried out to confirm specificity. Validation of Proteintech 19777-1-AP comprised of (1) Western blot of 3 different whole-cell lysates to show a single band at expected molecular weight; (2) peptide blocking, which eliminated the antibody reactivity and (3) short hairpin (sh)RNA knockdown in transfected HeLa cells, which showed a marked reduction in α_1A_-AR. These validation methods align with the published recommendations for assessment of specificity of antibodies [29].

### 4.4. Immunofluorescence on Human Tissue

Vascular α_1A_-AR expression was assessed in all cases using immunofluorescence and confocal microscopy. We first investigated α_1A_-AR expression with vessel type (capillaries, arteries or veins) and then cell-specific expression using markers for endothelium and smooth muscle cells. Sections were deparaffinised and rehydrated through xylene and a graded series of alcohols to PBS. Antigen retrieval was performed by microwaving in citrate buffer (10 mM, pH 6) for 25 min. After washing with PBS, sections were incubated in pepsin (1 mg/mL dissolved in 0.2 M HCL) at 37 °C for 3 min. Sections were washed three times with PBS and blocked with 15% normal goat serum diluted with PBSt for 1 h. Sections were incubated overnight with anti-SMA (mouse monoclonal, 1:200, Millipore), anti-α_1A-_AR (Rabbit polyclonal, 1:200, ProteinTech 19777-1-AP) and Dylight 647 tagged endothelium marker; Lycopersicon Esculentum (Tomato) lectin (1:100, Vector DL1178) at 4 °C. After washing with PBS, sections were incubated with biotinylated goat anti-rabbit (1:200, Vector) and goat anti-mouse AlexaFluor 594 conjugated (1:200, ThemoFisher A11032) secondary antibodies for 1 h at room temperature. Sections were washed again with PBS before incubation with streptavidin 488 (Vector) for 1 h at room temperature. Slides were washed with PBS and incubated with Sudan Black (Sigma-Aldrich, 1% in 70% alcohol) for 3 min to quench autofluorescence. Sections were rinsed with PBS and mounted with Mowiol (Sigma-Aldrich).

In order to ascertain whether α_1A_-AR were preserved in vessels exhibiting Aβ deposition in the vessel wall (CAA), double immunostaining for α_1A_-AR and Aβ was performed on the cases with CAA. Sections were deparaffinised and rehydrated through a graded series of alcohols. Sections were washed three times with tris-buffered saline (TBS) and incubated in 98% formic acid for 1 min at room temperature. Antigen retrieval was performed by microwaving in citrate buffer for 25 min. After washing with TBS, sections were first incubated in pepsin (1 mg/mL dissolved in 0.2 M HCL) at 37 °C for 3 min and then in a blocking solution consisting of TBS with 0.1% Triton and 15% normal goat serum (Sigma) for 1 h prior to incubation with primary antibodies. Sections were incubated overnight with anti-amyloid beta (mouse monoclonal isotype IgG2b, 1:100, (Biolegend SIG-39220)) and anti-α_1A_ (rabbit polyclonal, 1:200, (ProteinTech 19777-1-AP)) at 4 °C. After washing with TBS, sections were incubated in secondary antibodies comprising Alexa fluor 647 goat anti-mouse IgG2b (1:200, Invitrogen A-21242) and Alexa Fluor 555 goat anti-rabbit (1:200 Invitrogen A-21429) for 1 h at room temperature. Slides were washed in TBS and incubated with Sudan Black (Sigma-Aldrich, 1% in 70% alcohol) for 3 min to quench autofluorescence before being washed in TBS and coverslipped with Mowiol (Sigma-Aldrich).

### 4.5. Immunofluorescence on Cell Cultures

After 72 h in culture, cells were fixed with 4% paraformaldehyde for 10 min at room temperature, rinsed thoroughly with PBS and then blocked with 15% normal goat serum diluted in PBSt for 1 h before overnight incubation with anti-α_1A_-AR (Rabbit polyclonal, 1:200, ProteinTech 19777-1-AP) primary antibody. Cells were further rinsed with PBS before incubation with goat anti-rabbit Alexa Fluor 488 conjugated secondary antibody (Invitrogen, A-11034) for 1 h, followed by incubation in 2 µg/mL 4′,6-diamidino-2-phenylindole (DAPI) (Thermo Fisher, D1306) for 10 min. Cells were rinsed with PBS, and then coverslips were carefully removed from the 24-well plate and mounted onto slides with Mowiol and CitiFluor.

### 4.6. Imaging

The DAB-stained occipital lobe sections were imaged with an Olympus dot slide microscope. Using the tissue microarray function, ten adjacent non-overlapping images of 0.5 mm^2^ were obtained from the grey matter and the underlying white matter of each case using the 10× objective. Blood vessels that were observed in the grey matter, white matter and leptomeninges were imaged with the 20× objective. To prevent selection bias, the first five blood vessels observed in each region of interest were imaged, regardless of size, shape or plane. Representative images from the immunofluorescence staining were captured using a Leica TCS SP8 laser scanning confocal microscope.

### 4.7. Image Analysis and Statistics

The images obtained from the Olympus dot slide were used to quantify the expression of α_1A_-AR in the human brain sections. The percentage area stained for each image was calculated using an automated macro created using the ImageJ software, and an average was calculated from the values obtained from the ten images. To calculate the percentage area of staining in the vessel wall, ImageJ software was used to draw around the vessel, and the area stained was divided by the total area to give a percentage staining in the vessel wall. Where a transverse section of a blood vessel was captured, the lumen area was subtracted from the total area before dividing by the area stained. An average was obtained from the five vessels per region per case. Data were exported to SPSS software version 25 for statistical analysis. A Pearson’s bivariate correlation analysis was carried out to assess the relationship between adrenergic receptor staining and post mortem delay. A sensitivity test was also performed to investigate potential outliers. Percentage area stained data were analysed using a one way ANOVA with Bonferroni post hoc correction for multiple comparisons. Data are presented as mean ±95% confidence intervals with differences considered significant if *p* < 0.05.

## Figures and Tables

**Table 1:** Percentage area stained for overall and vascular specific α_1A_ adrenergic receptor in grey, white and leptomeninges per disease state and the comparisons between disease states.

**a**		**Percentage Area Stained for Overall α_1A_ Adrenergic Receptor in…**											
		**Grey Matter**			**White Matter**								
	**Disease state**	**N**	**Mean (SD)**	**(Min, Max)**	**N**	**Mean (SD)**			**(Min, Max)**				
	**Young (Y)**	5	3.56 (1.18)	(2.68, 5.62)	5	1.47 (0.47)			(1.11, 2.14)				
	**Old Non-demented (O)**	5	2.48 (0.97)	(1.48, 3.97)	5	0.79 (0.49)			(0.38, 1.48)				
	**CAA (C)**	5	1.68 (0.71)	(0.59, 2.56)	5	0.91 (0.41)			(0.39, 1.53)				
**b**		**Grey Matter**			**White Matter**								
	**Multiple Comparisons between Disease States**	**N**	**Mean Difference (95% CI)**		***p*^1^**		**N**	**Mean Difference (95% CI)**		***p*^1^**			
	**O–Y**	10	−1.09 (−2.79, 0.62)		0.305		10	−0.68 (−1.49, 0.13)		0.113			
	**C–Y**	10	−1.88 (−3.59, −0.18)		0.029		10	−0.56 (−1.37, 0.25)		0.233			
	**C–O**	10	−0.79 (−2.50, 0.91)		0.658		10	0.12 (−0.69, 0.93)		1.000			
**c**		**Percentage Area Stained for Vascular Specific α_1A_ Adrenergic Receptor in…**											
		**Grey Matter**			**White Matter**					**Leptomeninges**			
	**Disease State**	**N**	**Mean (SD)**	**(Min, Max)**	**N**	**Mean (SD)**			**(Min, Max)**	**N**		**Mean (SD)**	**(Min, Max)**
	**Young (Y)**	5	23.25 (3.33)	(17.56, 25.78)	5	17.58 (9.84)			(8.84, 34.09)	5		13.33 (4.21)	(6.81, 17.99)
	Old **non-demented (O)**	5	23.22 (5.42)	(15.40, 29.40)	5	15.67 (6.81)			(7.21, 21.89)	5		20.82 (10.03)	(10.67, 34.60)
	**CAA (C)**	5	18.81 (3.71)	(15.54, 24.99)	5	15.72 (6.75)			(7.02, 23.12)	5		13.93 (2.91)	(10.71, 18.22)
**d**		**Grey Matter**			**White Matter**					**Leptomeninges**			
	**Multiple Comparisons between Disease States**	**N**	**Mean Difference (95% CI)**		***p*^1^**		**N**	**Mean Difference (95% CI)**		***p*^1^**	**N**	**Mean Difference (95% CI)**	***p*^1^**
	**O–Y**	10	−0.03 (−7.51, 7.44)		1.000		10	−1.91 (−15.86, 12.05)		1.000	10	7.50 (−3.93, 18.93)	0.280
	**C–Y**	10	−4.44 (−11.92, 3.03)		0.373		10	−1.86 (−15.81, 12.09)		1.000	10	0.61 (−10.82, 12.04)	1.000
	**C–O**	10	−4.41 (−11.88, 3.06)		0.381		10	0.05 (−13.90, 14.00)		1.000	10	−6.89 (−18.32, 4.54)	0.359

^1^*p*-values are adjusted for multiple comparisons using Bonferroni correction; significance level is set at *p* < 0.050.

**Table 2:** Demographics for the cases used.

Source	Age	Sex	pm Delay/Hrs	Category
Edinburgh	51	M	78	Young
Edinburgh	41	F	50	Young
Edinburgh	60	M	52	Young
Edinburgh	59	F	53	Young
Edinburgh	33	M	47	Young
Newcastle	73	M	25	Old non-demented
Newcastle	90	M	18	Old non-demented
Newcastle	95	F	66	Old non-demented
Newcastle	95	M	21	Old non-demented
Newcastle	89	F	98	Old non-demented
Newcastle	67	M	46	CAA
Newcastle	86	F	51	CAA
Newcastle	73	M	7	CAA
Newcastle	74	F	49	CAA
Newcastle	87	F	54	CAA
